# The Action of King Edward's Hospital Fund for London

**Published:** 1919-04-12

**Authors:** 


### PENSIONS FOR HOSPITAL WORKERS.

### The Action of King Edward's Hospital Fund for London.

But for the war, which has brought about many important alterations in the conditions of service of hospital workers, it is probable that a scheme for providing pensions in old age or on retirement through disablement would before now have been instituted in respect of the voluntary hospitals, or at least those of them which are situated in the London district.

The Hospital Officers' Association, a body of workers in hospitals such as secretaries, dispensers, x-ray operators, inquiry officers, and the like, had appointed some six or seven years ago a committee to inquire into existing Pension Funds in connection with hospitals, and the practice of hospitals generally in respect of pensions. This committee having reported, the Association appealed to King Edward's Hospital Fund for London to take the matter into their consideration.

The King's Fund at once inquired of the committees of the voluntary hospitals within the Metropolitan area as to whether they would favourably consider a scheme if it were brought forward. The answer being generally in the affirmative, the Fund appointed a sub-committee consisting of Mr. W. J. H. Whittall, F.I.A., F.A.S. (Chairman), Sir William J. Collins, K.C.V.O., M.D., F.R.C.S., M.P., and Mr. Henry L. Hopkinson, with the following terms of reference:

"To inquire and report as to the existing provision for pensions to officers and staff employed in the London voluntary hospitals, and as to what alteration or extension (if any) of the existing arrangements is desirable and practicable, and generally to make such recommendations as they may consider advisable."

This Committee proceeded to examine the matter in all its details and aspects, and the result of their labours is the issue of the report of 273 foolscap pages, which has now been distributed by King Edward's Hospital Fund.* This report, but for the absorbing call upon everybody's time by the manifold duties caused by the war, would have been issued much earlier.

The report, in the nature of things, deals wholly with the hospitals of London, in the sense at present adopted by the Distribution Committee of the Fund—viz., the area comprised within the County of London or within nine miles of Charing Cross. The preface says, however, that it seems obvious, other things being equal, that a scheme capable of extension to or interchange with other hospitals would offer greater advantages.

The inquiry naturally resolved itself into three parts—viz., what is being done, what it is possible to do, and what it is advisable to do.

### Existing Pension Arrangements.

So far as regular pension schemes are concerned, little is done in respect of the 105 hospitals from which particulars were obtained by the Sub-Committee. There are, however, six hospitals—viz., the London, St. Bartholomew's, Guy's, the Middlesex, St. George's, and the Cancer Hospital—all institutions where, it may be said, there are substantial endowments or large invested funds, where general pension schemes exist. In all cases the plan is of the same type as that of the Civil Service pension scheme under the Superannuation Act of 1859 before the modification introduced in 1909. The pensions are non-contributory, and are calculated at a certain fraction—usually one-sixtieth—of the final salary for each year of service, up to a maximum of two-thirds of the final salary. There are varying conditions in respect of compulsory retirement, breakdown in health, and other matters.

In the remaining hospitals, which represent about 95 per cent. of the hospitals of London, there are no pension schemes, but the Sub-Committee has received no evidence of any case where a pension has been refused to a deserving person after long service. The drawbacks to a haphazard method of dealing out pensions are fully illustrated by the examples given of pensions paid by the hospitals without schemes. We have several illuminating instances, such as a case of a secretary retiring through ill-health after thirty years of service, his final salary being £600 and his pension £300; and one of another secretary retiring at the same age and after the same length of service with a final salary of £400 and receiving a pension of £75. The last case, unless there are unknown circumstances, may be quoted as illustrating the insecurity of an officer who is dependent in the matter of his pension upon such adventitious factors as the personnel of the Board in the year of retirement, the question of the finances of the hospital round about that period, and the like.

In respect of hospitals with schemes, some instances are given as follows:

Secretary, age 65, 45 years of service, final salary £750, pension £550.

Engineer, age 61, 41 years of service, final salary £152, pension £92.

### The Question of Migration.

But whether a hospital has a scheme or deals with each case on its merits the important point of loss of pensionable service through migration often arises. It is recognised that at present the worker moving from one hospital to another loses in practically every case. This aspect of the question is reviewed at length in the report, and it is concluded:

(a) That the absence of a comprehensive pension scheme does act as a penalty on migration, and, therefore, discourages migrating.

(b) That the principle of migration in analogous service is, on the whole, beneficial alike to the institutions and to the officers concerned, and should be encouraged instead of discouraged.

(c) That the small hospitals experience the resultant difficulties much more than the large.

### Special Features affecting Pensions for Nurses.

One of the special features of the problem, when it touches upon the nursing staff of a hospital, is the fact that a very large proportion of the nursing staff at any given time usually consists of probationers or of partially trained or newly qualified nurses, who spend only the early years of their profession and career in the service of a particular hospital, and who very frequently pass sooner or later out of hospital service altogether and into private nursing. Another, is the existence of a special fund, the Royal National Pension Fund for Nurses, established for the express purpose of providing, by means of mutual insurance, for the pension needs of nurses, whether engaged on hospital work or not. With

* Published by C. & E. Layton, 56 Farringdon Street, Holborn, E.C. 4.

**Pensions for Hospital Workers**—(*continued*).

nurses generally the tendency to change often continues throughout their career, even with those who return later to hospital nursing. The result is that at a hospital, the qualified staff, at all events below the rank of matron and sister, most often consists largely of nurses who have been only a comparatively short time in the service of the institution and who may not remain in its service long.

There are eight hospitals which have schemes of their own for the provision of pensions for the nursing staffs—namely, the six mentioned in the early part of this article and Poplar Hospital and Cheyne Hospital for Children. There are seven others which are affiliated with the Royal National Pension Fund for Nurses—namely, the Dreadnought, the Hospital for Consumption (Brompton), King's College Hospital, Paddington Green, Queen Charlotte's, the Royal Free, and St. Mary's. Guy's has a combined scheme of affiliation and direct pensions.

The general conclusion in the report is that the existing provision for pensioning the nursing staffs is very insufficient and unsatisfactory, but that a distinction must be drawn between the cases of those matrons, sisters and some staff nurses, the nature and duration of whose service is comparable to that of hospital officers, and the case of those nurses and probationers the nature and duration of whose service is so different from that of other hospital officers as to call for different treatment in the matter of pensions.

Various Schemes Surveyed.

Part II. of the report deals with what it is possible to do, by means of a survey of various methods of providing pensions, and for the purpose proceeds to examine typical schemes already in operation in certain Government, municipal, railway, educational, hospital and other services. It may be said here that this part of the report constitutes a document in which facts are ably marshalled in a form which could very well provide a useful guide to anybody, whether a hospital or not, which is considering a pension scheme.

The large mass of information thus compiled lends valuable help in elucidating the fundamental questions that are inseparable from a scheme for hospital workers. It is to be determined whether the pensions should be wholly or partly provided for before they fall due, whether death benefits as well should be provided, whether the workers themselves should contribute, whether the pensions should be based on unknown future salaries, or, as adopted by the Royal National Fund for Nurses, should assure definite amounts, whether existing officers can be included, whether it would be possible to give earlier pensions in the event of disablement through loss of health, and whether and by what means it would be possible to reconcile the grant of pensions by a central body with the independence and freedom of internal administration to which the hospitals attach much importance. The answer to these questions in the light of the working of other funds is that pensions should be provided wholly in advance, death benefits should be provided, workers should contribute and pensions must represent an adequate proportion of the salary, disablement should be provided for, and existing officers should be included.

Final Conclusions and Recommendations.

The Committee, having examined with great care the problem as to under what financial system a fund should be established, come to the conclusion that a scheme based upon recourse to insurance companies is the only possible solution of the superannuation problem before them. They recommend that a joint conference of hospital representatives should be invited to consider the whole question on that basis. In addition to the hospital committees and officers, care should be taken that the nursing staffs and weekly wage class are separately represented. Experimental calculations should be based on the assumption that the employer and the beneficiary should each contribute 5 per cent of the salary, so as to make a total available contribution of not less than 10 per cent. for purchasing the benefits. The system should be made obligatory in the case of all new appointments, so that all officers coming into the scheme would be consenting parties to the contribution.

The question of including the existing staffs is a question of great cost if full benefits are to be provided in advance for them. It is in these cases recommended that future years should be treated as pensionable and that it be left to hospital committees to make supplementary payments in arrear to increase the security of the officers in question. It would further be one of the first tasks of any general Council administrating the scheme to direct an actuarial investigation into the cost of including the existing staffs in respect of past service, and generally to consider the question of raising a special fund.

It is further recommended that the King's Fund shall take into consideration the best way of making provision in advance for disablement benefits by means of a central body and offer its help by way of advice, or, if thought fit, of pecuniary assistance.

The report is signed by Mr. W. J. H. Whittall and Mr. Henry L. Hopkinson.

A Dissentient Memorandum.

Attached to the report is a dissentient memorandum by Sir William Collins, who is unable to agree with his colleagues in respect of the only solution being a scheme based on insurance, and does not consider that any conference of hospital representatives should be exclusively limited in its deliberations by this condition. Sir William agrees that the case submitted by the Hospital Officers' Association has, generally speaking, been made out; he also believes that the prejudice against making provision, out of the funds of voluntary charity, for the old age of an officer who has rendered long and faithful service has largely, if not wholly, disappeared. He suggests that, having regard to the fact that the provision of pensions is regarded by King Edward's Hospital Fund as legitimate expenditure of ordinary hospital revenue, towards which the fund makes large subventions, and to the fact that by its statutory powers it is a legitimate use of its general or special funds to render financial support, whether by guarantee or subvention, towards securing adequate pensions for hospital officers or staffs, there is no insuperable objection to the Fund rendering assistance to the hospitals in this respect.

Seeing that the adoption alike of a "Mutual Principle Scheme" and an "Insurance Company Scheme" appears to require the administrative and financial aid of a central body in which the hospitals have confidence, and seeing that the King's Fund is a body that, if willing, is able to provide such requirements, Sir William Collins cannot agree that an insurance scheme, supplemented as suggested by a subsidised scheme for disablement benefits, is the only possible solution of the problem.

The members of the Committee record their acknowledgments of the services rendered by Mr. H. R. Maynard, the secretary of King Edward's Hospital Fund, and of Mr. T. Tinner, F.I.A., and Mr. F. L. Collins, F.I.A., the two actuaries who contributed largely to the material contained in the report.

